# Production of GMP-Compliant Clinical Amounts of Copper-61 Radiopharmaceuticals from Liquid Targets

**DOI:** 10.3390/ph15060723

**Published:** 2022-06-07

**Authors:** Alexandra I. Fonseca, Vítor H. Alves, Sérgio J. C. do Carmo, Magda Silva, Ivanna Hrynchak, Francisco Alves, Amílcar Falcão, Antero J. Abrunhosa

**Affiliations:** 1 ICNAS Produção Unipessoal, Lda., Ed. ICNAS, Polo das Ciências da Saúde, University of Coimbra, 3000-548 Coimbra, Portugal; alexandrafonseca@icnas.uc.pt (A.I.F.); vitoralves@uc.pt (V.H.A.); sergiocarmo@uc.pt (S.J.C.d.C.); magdasilva@icnas.uc.pt (M.S.); ivanna.ua@icnas.uc.pt (I.H.); amilcar.falcao@uc.pt (A.F.); 2 Fluidomica, Lda., 3060-197 Cantanhede, Portugal; 3 CIBIT/ICNAS, Coimbra Institute for Biomedical Imaging and Translational Research/Institute for Nuclear Sciences Applied to Health—University of Coimbra, 3000-548 Coimbra, Portugal; franciscoalves@uc.pt; 4 Instituto Politécnico de Coimbra, ESTeSC—Coimbra Health School, 3040-854 Coimbra, Portugal; 5 Faculty of Pharmacy, University of Coimbra, 3000-548 Coimbra, Portugal

**Keywords:** radiometals, copper-61, liquid targets, post-processing, [^61^Cu]Cu-DOTA-NOC, [^61^Cu]Cu-DOTA-TOC, [^61^Cu]Cu-DOTA-TATE

## Abstract

PET imaging has gained significant momentum in the last few years, especially in the area of oncology, with an increasing focus on metal radioisotopes owing to their versatile chemistry and favourable physical properties. Copper-61 (t_1/2_ = 3.33 h, 61% β^+^, E_max_ = 1.216 MeV) provides unique advantages versus the current clinical standard (i.e., gallium-68) even though, until now, no clinical amounts of ^61^Cu-based radiopharmaceuticals, other than thiosemicarbazone-based molecules, have been produced. This study aimed to establish a routine production, using a standard medical cyclotron, for a series of widely used somatostatin analogues, currently labelled with gallium-68, that could benefit from the improved characteristics of copper-61. We describe two possible routes to produce the radiopharmaceutical precursor, either from natural zinc or enriched zinc-64 liquid targets and further synthesis of [^61^Cu]Cu-DOTA-NOC, [^61^Cu]Cu-DOTA-TOC and [^61^Cu]Cu-DOTA-TATE with a fully automated GMP-compliant process. The production from enriched targets leads to twice the amount of activity (3.28 ± 0.41 GBq vs. 1.84 ± 0.24 GBq at EOB) and higher radionuclidic purity (99.97% vs. 98.49% at EOB). Our results demonstrate, for the first time, that clinical doses of ^61^Cu-based radiopharmaceuticals can easily be obtained in centres with a typical biomedical cyclotron optimised to produce ^18^F-based radiopharmaceuticals.

## 1. Introduction

The use of advanced imaging technologies, especially nuclear medicine (i.e., PET and SPECT), can enhance diagnosis, staging, treatment planning and evaluation of treatment response in cancer care. Over the last two decades, an emerging quantity of small biomolecules (e.g., peptides, antibodies, antibodies fragments or nanoparticles) have been labelled with beta- or alpha-emitting metal radionuclides (e.g., gallium-68, copper-64, luthetium-177, actinium-225 and astatine-211) for imaging and therapeutic applications [1,2,3,4,5]. The wide variety of physical decay properties and half-lives, the simple and fast one-step radiolabelling chemistry [6,7]—easily adaptable to any type of vector for any target delivery—and the easy translation of metal-based radiopharmaceuticals into a theranostic approach [8] have primarily contributed to their interest and, currently, are major contributors to its success.

^68^Ge/^68^Ga generators play a substantial role in this growing phenomenon by allowing worldwide access to gallium-68 (t_1/2_ = 68 min, 89% β^+^, E_max_ = 1.899 MeV)—even in small hospital radiopharmacies (not requiring an onsite cyclotron)—which simplifies the translation of ^68^Ga-conjugated peptides from the bench to routine clinical use [9]. Notwithstanding this, because of the worldwide shortage of gallium-68 generators, they are gradually losing out to more cost-effective production methods (i.e., accelerator-produced radiometals by the irradiation of natural or enriched targets). These methods are able to produce higher amounts of activity, without waiting time between productions (unlike the typical 3–4 h interval between elutions of ^68^Ge/^68^Ga generators), aiming at fulfilling the ever-increasing clinical needs of gallium-68 [10,11]. The recently approved monograph of gallium-68 chloride solution produced from zinc-68 irradiation (Eur. Ph. 3109) [12] is a clear sign of the need for accelerator-produced methods in radiochemistry centres worldwide, which have access to this technology. Furthermore, since ^68^Ge/^68^Ga generators are no longer a discriminatory advantage for using gallium-68 over other metal radionuclides on a routine basis, new promising radionuclides with better physicochemical properties are arising (e.g., scandium-43, scandium-44, copper-61, copper-64 and zirconium-89) with significant advantages over gallium-68: (1) easier distribution to centres that do not have onsite cyclotrons, (2) lower maximum positron emission energies that meet the requirements for a new generation of tomographs with higher resolution and (3) nuclides having a close therapeutic match, which is determinant for personalised medicine as we enter the theranostic era [13,14].

Copper-61 (t_1/2_ = 3.33 h, 61% β^+^, E_max_ = 1.216 MeV) [15] is a positron-emitting radionuclide presenting decay characteristics comparable to gallium-68 but with the advantage of presenting lower maximum positron energy (E_max_ = 1.216 MeV vs. E_max_ = 1.899 MeV) and a substantially more practical half-life (3.33 h vs. 68 min). In the past few years, several groups have attempted to find the best production and purification methods for cyclotron-produced copper-61. Liquid and solid target irradiations have both been explored. In 2012, the production of copper-61 from natural cobalt solid targets, following the ^nat^Co(α,xn)^61^Cu nuclear reaction, resulted in high-purity copper-61 [16]. Later, Asad et al. [17,18] and Thieme et al. [19] detailed the production of copper-61 from natural zinc and zinc-64, also in solid targets. In 2017, our group described the production of copper-61 from the irradiation of liquid targets at low proton energies [20] and later its automated purification [21]. More recently, the possibility of producing copper-61 from solid natural nickel targets, following ^nat^Ni(d,x)^61^Cu nuclear reaction, was also outlined, along with its fully automated purification process [22]. Despite increasing efforts being made in the development of the above-mentioned methods towards being capable of producing high-purity copper-61, to date, only a handful of molecules have been labelled with this radioisotope—mostly thiosemicarbazone-based molecules (i.e., ATSM, PTSM, APTS and TATS) [23,24,25,26,27], which are well known for presenting high affinity for copper.

Considering the above, the aim of the current work is to demonstrate that the production of chelator-based copper-61 labelled radiopharmaceuticals can easily be performed and can be made Good Manufacturing Practise (GMP)-compliant for routine clinical use. For that purpose, we present the production, synthesis and quality control of [^61^Cu]Cu-DOTA-TATE, [^61^Cu]Cu-DOTA-TOC and [^61^Cu]Cu-DOTA-NOC, the copper-61 equivalents of the somatostatin (SST) analogues extensively used with gallium-68, in current clinical practice [28].

Initial work on targeting and staging neuroendocrine tumours (NETs) through the labelling of SST analogues begun with [^123^I]I-Tyr3-octreotide [29]. The first Food and Drug Administration (FDA)-approved radiopharmaceutical was Octreoscan^®^, in 1994 ([^111^In]In-DTPA-Octreotide) [30,31]. Today, ^68^Ga-labelled radiopharmaceuticals such as [^68^Ga]Ga-DOTA-NOC, [^68^Ga]Ga-DOTA-TOC and [^68^Ga]Ga-DOTA-TATE are in current clinical practice to diagnose, with PET, solid tumours which over-express SST receptors (SSTRs) [32,33]. The ready-to-label “cold kit” with DOTA-TATE was approved by the FDA in 2016 (Netspot^TM^), and the equivalent with DOTA-TOC (Somakit-TOC) was approved by the EMA in 2017 [34].

Most previous works regarding the labelling of SST analogues with copper focused on copper-64 (t_1/2_ = 12.7 h, 18% β^+^, E_max_ = 0.653 MeV). A first-in-human study with [^64^Cu]Cu-DOTA-TATE revealed several advantages, i.e., higher lesion detection, better image quality and lower radiation doses, when compared with [^111^In]In-DTPA-octreotide when used for SPECT imaging [35]. More recent clinical studies, particularly head-to-head comparisons of [^64^Cu]Cu-DOTA-TATE with both [^111^In]In-DOTA-TATE [36] and [^68^Ga]Ga-DOTA-TOC [37] revealed an overall better performance of the ^64^Cu-conjugated in terms of sensitivity, resolution and rate of lesion detection. Additionally, other first-in-human studies with [^64^Cu]Cu-DOTA-TOC also showed high lesion detection rate, safety of use and high effectiveness for predicting treatment planning [38]. Even more recently, Loft et.al. confirmed the extended imaging window of [^64^Cu]Cu-DOTA-TATE from 1 h to 3 h [39], without a decrease in performance. There is still work in progress aiming at clarifying the dosimetric parameters and predicting both the overall survival (OS) and progression-free survival (PFS) ability of these radiopharmaceuticals labelled with copper-64 [40,41,42]. These results lead us to conclude that the substitution of gallium for copper on these SST analogues has most likely a positive impact on their performance as PET radiopharmaceuticals. Moreover, the favourable physical properties of copper-61 when compared with copper-64 (shorter half-life, higher β^+^-emission) makes it an ideal nuclide for this purpose.

In this context, the simple, cost-effective production and separation methods herein described could pave the way for the widespread clinical use of copper-61 radiopharmaceuticals, providing an even better alternative to the scarce and expensive-to-obtain gallium-68.

## 2. Results and Discussion

### 2.1. [^61^Cu]CuCl_2_ Production

Copper-61 was produced using the target system previously described in [20] and then in [43]. Several cyclotron irradiations were performed with both natural and enriched zinc. Table 1 summarises the number of runs, the irradiation conditions and the activity produced for each target. The same solution of zinc-64, with an initial concentration of 200 mg/mL, was irradiated a maximum of four times.

A direct comparison between the 180 min long irradiation of natural zinc and first-time irradiated enriched zinc-64 showed that, under the same irradiation conditions, i.e., time, concentration, current and pressure, the use of zinc-64 allowed the production of twice the activity of copper-61 than natural zinc: 3.65 ± 0.18 GBq (N = 8) and 1.84 ± 0.24 GBq (N = 20), respectively. These correspond to low yields when compared to solid targets, as stated in [44]; however, the latter also come with high cost and tremendous operational complexity. These studies confirmed the expected higher activities of copper-61 from the enriched target, considering the 49.2% abundance of the zinc-64 isotope in the natural zinc. Although higher activities of copper-61 are produced using enriched target material, zinc-64 is approximately 200 times more expensive than the natural target (550–669 €/g zinc-64 vs. 2.92 €/g natural zinc). Given this tremendous difference, a cost–benefit analysis is required.

### 2.2. Recovery and Recycling of ^64^Zn

One of the advantages of using liquid targets is that the recycling of enriched material is simplified. This is especially important when considering the high cost of zinc-64. Notwithstanding, few authors have actually described this. In this study, the zinc-64 target was recovered from the CU resin waste container several days after been irradiated. It was then evaporated and re-dissolved into the initial form of 10 mM HNO_3_. Moreover, the recycling process was simple, since no solvent other than HNO_3_ was introduced during the purification process, and the zinc-64 solution could be re-used directly after filtration. The percentage of zinc-64 recovered and re-irradiated was collectively determined to be higher than 90% each time it was recycled. We found a slight variation in the activity produced (corrected at EOB), depending on how many times the zinc-64 solution was recycled and subsequently irradiated (Table S1). The second irradiation of the same batch of zinc-64 did not show a significant decrease in the amounts of produced nor purified copper-61. On the other hand, with more than two irradiations, there was a statistically significant decrease in the amount of copper-61 produced and, consequently, in purified copper (Table S1). This decrease in the activity of copper-61 is explained by the loss of zinc-64 during the several steps of the process: recycling, purification, evaporation and final filtration of the solution. Regarding isotopic enrichment of the recycled solutions (Table S1), the recovery process did not lead to a significant decrease in zinc-64 enrichment.

The purification process was performed as described earlier [45] without further modifications.

### 2.3. [^61^Cu]Cu-DOTA-NOC, [^61^Cu]Cu-DOTA-TATE and [^61^Cu]Cu-DOTA-TOC Production Activity Distribution

[^61^Cu]Cu-DOTA-NOC, [^61^Cu]Cu-DOTA-TOC and [^61^Cu]Cu-DOTA-TATE were produced using the Synthera^®^ Extension automated module (IBA, Louvain-la-Neuve, Belgium). This fully automated process complies with GMPs to produce radiopharmaceuticals (EudraLex, Volume 4, Annex 3) (i.e., the use of disposable cassettes and tubing systems, ensuring high quality and reproducibility of the final radiopharmaceutical product and the narrowing of the risk of radioactive cross-contamination). A total of 50 μg of DOTA-NOC (N = 10), DOTA-TATE (N = 3) and DOTA-TOC (N = 3) were labelled with purified [^61^Cu]CuCl_2_ at a 85–100 °C reaction temperature and 10 min reaction time. Specifications are summarised in Table 2.

As expected, depending on the production route of [^61^Cu]CuCl_2_ used, the greatest difference found was in the amount of [^61^Cu]Cu-DOTA-NOC activity at the End Of Synthesis (EOS): 0.99 ± 0.16 GBq or 1.95 ± 0.21 GBq from natural or enriched targets, respectively. To evaluate the efficacy and reproducibility of this synthesis method, we determined the radiochemical and labelling yields of the process. Radiochemical Yield (RCY) refers to the final activity in the product of [^61^Cu]Cu-DOTA-NOC/TOC/TATE, expressed as the percentage (%) of starting activity of [^61^Cu]CuCl_2_ obtained after purification [46]. The quantity of both was decay-corrected to the same time point. All radioactivity lost during transfer, labelling reaction, solid phase purification (SPE) and dispensing were accounted for in the RCY. Whereas the labelling yield indicated the direct yield of the labelling reaction.

Nonetheless, labelling yield referred only to the extent of the labelling reaction, comparing the amount of [^61^Cu]CuCl_2_ that reacted into [^61^Cu]Cu-DOTA-NOC/TOC/TATE, and did not consider any other process losses. The data showed that neither labelling yields nor RCY were affected by the amount of activity, which confirms that activities of copper-61 up to 2.7 GBq (at the End Of Purification (EOP)) do not have negative effects on the synthesis process of these radiopharmaceuticals.

**Figure 1:**
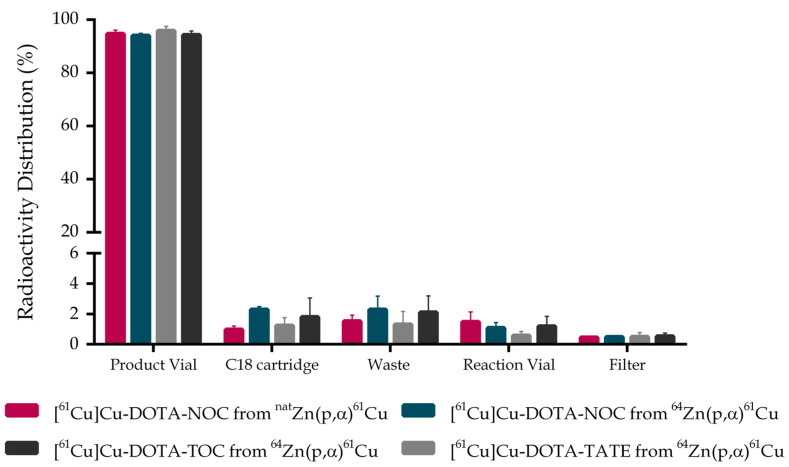
Activity distribution of the different cassette components after synthesis on Synthera^®^ Extension module: Final Product Vial, C18 SPE cartridge, Waste and Reaction vial. Data comprises the different radiopharmaceuticals produced (mean ± SD, N ≥ 3).

We also compared the distribution patterns regarding the different cassette components for all radiopharmaceuticals (Figure 1). Activity distribution revealed similar results for all peptides. It is important to note the low residual activity in the different components and the small SD of its values, which reflects both high reproducibility and efficacy of the automated synthesis process.

### 2.4. Quality Control

Table 3 outlines the final product specifications obtained, including radiochemical and radionuclidic purity, radionuclidic identity and pH. Radiochemical purity was evaluated by radio-HPLC, using the methods described in the next section (Table 4 in Materials and Methods). Radionuclidic purity was evaluated using a High Purity Germanium (HPGe) detector, several hours after EOS. A single value of radionuclidic purity is presented for [^61^Cu]Cu-DOTA-NOC, [^61^Cu]Cu-DOTA-TOC and [^61^Cu]Cu-DOTA-TATE produced from the enriched target, as radionuclidic purity is only dependent on the method of copper-61 production, regardless of the subsequent synthesis process.

**Figure 2:**
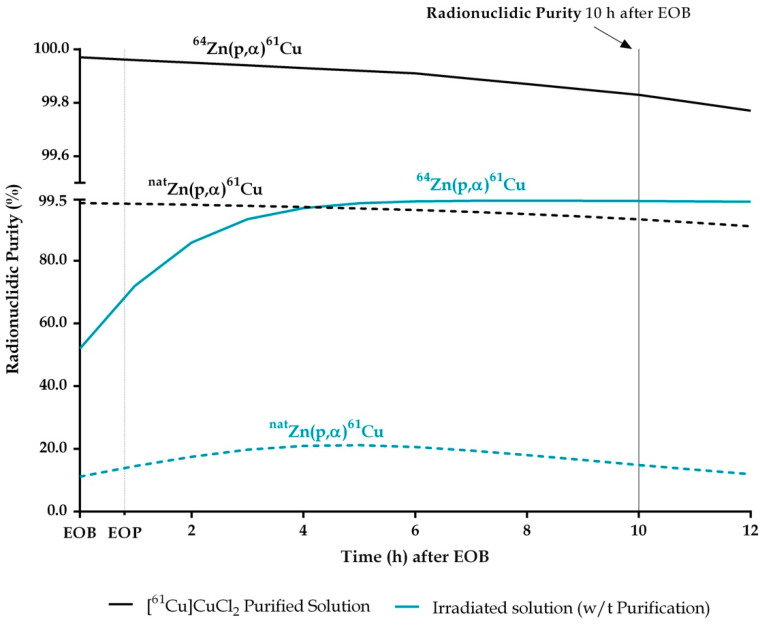
Radionuclidic purity (%) of copper-61 produced from liquid targets before purification (blue lines obtain from calculations [20]) and after purification (black lines obtain from HPGe measurements) either from natural Zinc (dashed lines) or enriched Zinc-64 (solid lines) experimental values determined by HPGe.

As expected, the choice of target material has an impact on radionuclidic purity. When copper-61 is produced from natural zinc, 1.5% of copper-64 (at EOB) is produced simultaneously with copper-61 [20], whereas when an enriched target is used, almost no copper impurities are expected to be produced; however, small amounts of copper-64 are present as a side product, resulting from the (p,α) nuclear reactions on residuals zinc-67 and zinc-68 present in the enriched target material. This amounts to about 0.03% of copper-64 co-produced when irradiating zinc-64. Figure 2 indicates the impact of this percentage of copper-64 on the shelf life of the product when each target is used.

Currently, no European Pharmacopeia (Ph. Eur.) monograph exists for copper-61 [47]. Taking into consideration the limits set for radionuclidic impurities in the Gallium (^68^Ga) Chloride (accelerator produced) monograph of 2% (mon. 3109), the production of copper-61 from natural Zinc would require it to be used immediately after purification. On the other hand, when produced from zinc-64, the radionuclidic purity of copper-61 is higher than 99% and remains at this level for many hours after production (Figure 2).

### 2.5. In Vitro Stability

**Figure 3:**
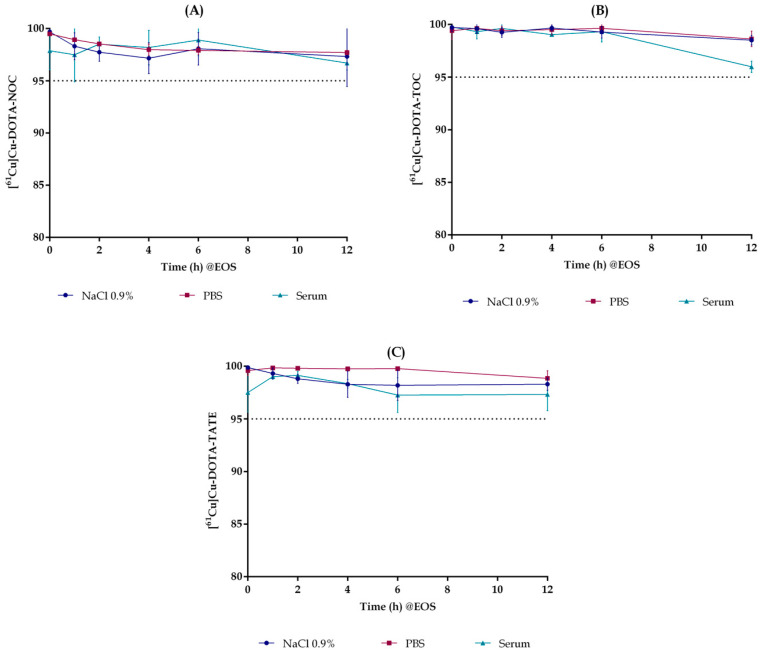
Stability of [^61^Cu]Cu-DOTA-NOC (**A**), [^61^Cu]Cu-DOTA-TATE (**B**) and [^61^Cu]Cu-DOTA-TOC (**C**) in NaCl 0.9%, PBS and mouse serum. Radiochemical purity results were obtained by radioHPLC at: T0, T0 + 1 h, T0 + 2 h, T0 + 4 h, T0 + 6 h and T0 + 12 h, where T0 represents the EOS.

The stability of [^61^Cu]Cu-DOTA-NOC, [^61^Cu]Cu-DOTA-TOC and [^61^Cu]Cu-DOTA-TATE in aqueous solution (NaCl 0.9% or PBS) and in mouse serum was evaluated up to 12 h after the EOS. Figure 3 shows that all radiopharmaceuticals were stable under the conditions tested. Radiochemical purity results indicated that these compounds are highly stable (over 95%) at 37 °C up to 12 h after the EOS, in the final formulation (NaCl 0.9%), PBS and serum.

## 3. Materials and Methods

All chemicals and solvents used for purification of [^61^Cu]CuCl_2_ and synthesis of ^61^Cu-conjugated peptides were trace metal grade, and HPLC solvents were HPLC grade. The remaining solvents and reagents (i.e., hydrochloric acid > 30% and nitric acid > 69% (Honeywell Fluka, Charlotte, NC, USA), bi-distilled water (BBraun, Melsungen, Germany), ethanol (Rotem, Israel), sodium acetate anhydrous (Honeyweell Fluka, Germany), *L*-ascorbic acid (Sigma-Aldrich, St. Louis, MO, USA) and DTPA (Alfa Aesar, Kandel, Germany)) were also trace metal basis, to prevent metal cross-contamination.

Zinc (99.998%) was acquired from Alfa Aesar, whereas the enriched zinc metal form (^64^Zn—99.89%) was obtained from CMR (Moscow, Russia). Purification and labelling disposable kits were purchased from Fluidomica (Cantanhede, Portugal) and purification resins (i.e., CU-B25-A resin and SAX 1 × 8 200–400 mesh, Cl^−^ form resin) from Triskem (Bruz, Belgium). Peptides DOTA-NOC acetate, DOTA-TATE acetate and DOTA-TOC acetate, fractioned and kept at −20 °C in an aqueous solution, were manufactured by ABX (Radeberg, Germany). The usage of polyethylene and polypropylene materials was favoured over that of glass materials.

### 3.1. Irradiation and Purification of [^61^Cu]CuCl_2_

Irradiation of zinc liquid targets, both natural and enriched, and further copper-61 purification was conducted following the previously published and described methodology [48,49]. Briefly, copper-61 was obtained from the irradiation of both highly pure zinc nitrate hexahydrate and enriched zinc-64 solutions using an IBA Cyclone 18/9 (IBA, Louvain-la-Neuve, Belgium) [20]. Zinc nitrate was directly dissolved in 10 mM nitric acid, yielding a concentration of 0.2 g/mL, whereas zinc-64 (metal form) had to be initially dissolved with highly concentrated nitric acid, left overnight, evaporated to dryness, and finally re-dissolved in 10 mM nitric acid, yielding likewise a concentration of 0.2 g/mL. Zinc-64 solutions were recycled and re-irradiated up to 4 times. Since only water and HNO_3_ were added to the original zinc-64 solution during the purification process, the recycling process was made possible simply by evaporating the excess of water. These solutions were irradiated at 65–75 μA for 180 min. Copper-61 automatic purification was conducted using a Synthera^®^ Extension module (IBA, Louvain-la-Neuve, Belgium) without any manual intervention, and it was completed in less than 40 min from the EOB [45].

### 3.2. EtOH as Radiolytic Scavenger

**Figure 4:**
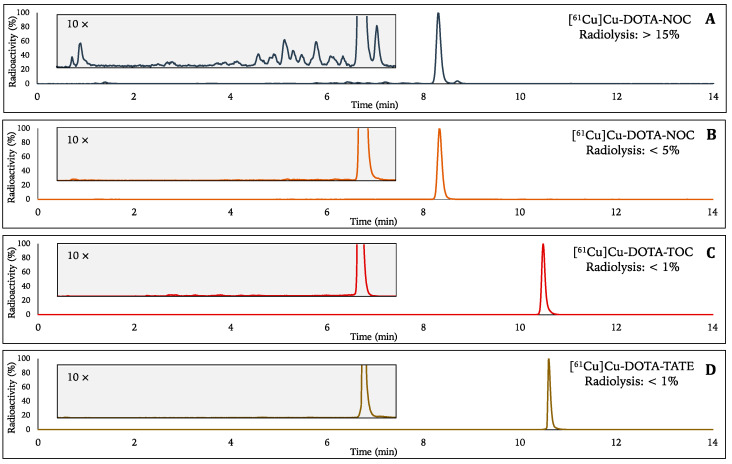
Representative chromatograms of [^61^Cu]Cu-DOTA-NOC, [^61^Cu]Cu-DOTA-TOC and [^61^Cu]Cu-DOTA-TATE without (**A**) and with (**B**–**D**) using EtOH (maximum 5 vol% EtOH) during the labelling reaction. Two different HPLC methods were used, as described in Table 4. For a more practical comparative analysis between chromatograms, raw data was normalised as percentage of total radioactivity. Percentage of radiolysis is the ratio of radiolysis counts to total counts.

The considerably high percentage of radiolysis in the final product vial (FPV), caused by the presence of free radicals in solution (e.g., superoxide or hydroxyl radicals) [50], impaired the establishment of the most favourable labelling conditions. Although not explicitly measured, for higher activity concentrations, the radiolysis percentage was anticipated to increase. Several compounds are known to act as radiolytic stabilizers and protect against radiolysis. Antioxidant compounds, such as ascorbic acid (AA) and gentisic acid (GA), are commonly known to protect against radiolysis and are mainly described in the literature for radiolabelling biomolecules with β^-^-emitting radionuclides (e.g., yttrium-90 and luthetium-177) [51,52]. Notwithstanding, these compounds might have a negative impact on copper-based radiopharmaceuticals, given the redox properties of copper. More recently, EtOH also gained relevance in ^68^Ga-based radiopharmaceuticals and proved to be of great value, as confirmed by Eppard et al. [53,54,55]. To evaluate the applicability of EtOH as a radiolytic scavenger for ^61^Cu-based radiopharmaceuticals, a single test with and without EtOH was performed before establishing optimal labelling conditions. Figure 4 shows the results attained when using EtOH up to 300 μL (maximum 5 vol% ethanol). Compared with the labelling of [^61^Cu]Cu-DOTA-NOC without EtOH (Figure 4A), it is evident that there is a significant decrease in the rate of radiolysis using the ethanol-based method (Figure 4B–D). This method showed that 5 vol% EtOH leads to a decrease in radiolysis from more than 15% in [^61^Cu]Cu-DOTA-NOC to less than 5% for [^61^Cu]Cu-DOTA-NOC and less than 1% for both [^61^Cu]Cu-DOTA-TOC and [^61^Cu]Cu-DOTA-TATE. We found that [^61^Cu]Cu-DOTA-NOC is the most sensitive peptide to radiolysis, even in the presence of EtOH. Based on these findings, the use of EtOH was implemented in all further labelling formulations to act as a radiolytic stabilizing agent during the labelling reaction.

### 3.3. Synthesis of [^61^Cu]Cu-Conjugated Peptides on the IBA Synthera^®^ Extension Module

**Figure 5:**
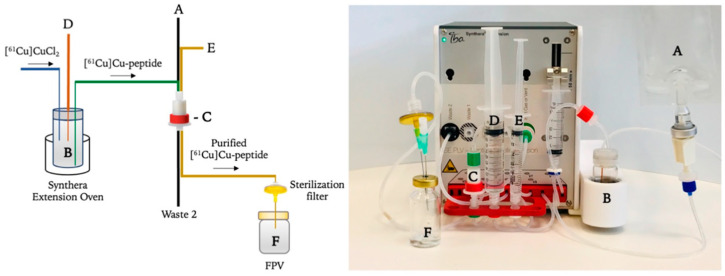
Schematic flow and Synthera^®^ Extension module device and disposable cassette: (A) Water, (B) Reaction vial and oven, (C) SPE C18 cartridge, (D) Buffer and peptide, (E) Ethanol/Water (50%/50%) solution and (F) product vial.

Fully automated post-processing synthesis was performed using a Synthera^®^ Extension module (Figure 5) and completed within a maximum of 25 min from the EOP, without any manual intervention. After the purification process, [^61^Cu]CuCl_2_ was automatically transferred to the reaction vial (B). Then, the peptide (DOTA-NOC acetate, DOTA-TOC acetate or DOTA-TATE acetate), dissolved in 2.5 M sodium acetate buffer, was transferred to the same reaction vial, where the reaction occurred. After the labelling reaction, the mixture was cooled down with water, and the product was then purified using a C18 cartridge (Sep-Pak Plus Short C18, Waters, Milford, Massachusetts, USA). After a rinse step, the ^61^Cu-conjugated peptide was eluted from the C18 cartridge with a mixture of (50/50%) water/ethanol.

The general automated synthesis/radiolabelling steps are as follows:

1. The C18 cartridge (C) is preconditioned with ethanol (10 mL) followed by water (10 mL) prior to use;
2. Purified [^61^Cu]CuCl_2_ (3 mL, 0.5 M HCl) is transferred to the reaction vial (B);
3. Peptide (50 μg), previously diluted in 2.5 M sodium acetate (3 mL) and EtOH (200–300 μL) (D) to prevent radiolysis, is transferred to the reaction vial (B) and mixed with [^61^Cu]CuCl_2_ for 10 s;
4. Radiolabelling reaction is conducted for 10 min, with variable temperature (85–100 °C) and pH fixed between 4 and 5;
5. Reaction mixture is cooled down with water (12 mL) (A) and passed through a C18 cartridge at 3 mL/min flow to the waste container (Waste 2);
6. C18 cartridge is then rinsed with water (10 mL) (A), which rinses the column at a 3 mL/min flow;
7. [^61^Cu]Cu-labelled peptide is finally eluted from the C18 column with a solution of water/EtOH (50/50%) (E) to the final product vial (F) with a 3 mL/min flow.

After labelling and purification, the FPV was transferred to the Quality Control (QC) laboratory, and all the components were measured, after which the radiochemical yield was determined.

### 3.4. Quality Control

#### 3.4.1. Radionuclidic Purity (HPGe)

The RNP of copper-61 at EOB was determined through γ-spectroscopy of the final solution using a High Purity Germanium detector (HPGe), several hours after the EOP. The HPGe was calibrated with ^154^Eu and ^133^Ba radioactive sources and placed in a low-background shielding. γ-spectra were acquired using point-source-like samples with a dead-time below 4%. GammaVision (ORTEC Inc., Easley, SC, USA) software was used to determine photopeak areas.

#### 3.4.2. Radiochemical Purity (Radio-HPLC)

RCP was measured by HPLC (Agilent 1200 series HPLC system, Agilent Technologies, Santa Clara, CA, USA) equipped with a GABIStar NaI(Tl) radiometric detector (Raytest Isotopenmessgeraete GmbH, Straubenhardt, Germany) (20 μL sample volume). Two different methods (Table 4) were used, one for [^61^Cu]Cu-DOTA-NOC (Method A) and a second to evaluate both [^61^Cu]Cu-DOTA-TATE and [^61^Cu]Cu-DOTA-TOC (Method B). An ACE 3 C18 150 × 3 mm HPLC column (ACE, Reading, UK) was used in both methods, and the flow was fixed at 0.6 mL/min.

#### 3.4.3. Stability Experiments

The stability of ^61^Cu-conjugated peptides was evaluated under various conditions: in the final formulation (10% EtOH/0.9% NaCl), in the presence of PBS and in mouse serum. All stability measurements were quantified by HPLC, as incubation solutions could affect the accuracy of Thin Layer Chromatography (TLC). The HPLC methods used to evaluate stability were previously described (in Table 4), with exception of [^61^Cu]Cu-DOTA-NOC. In this case, stability was evaluated using a faster method, with the following gradient: 0–5 min Mobile Phase A (100% to 0%).

#### 3.4.4. Stability in Aqueous Solvents

The published protocol was followed with minor changes [56]. Briefly, 50 μL of the final purified solution (Water/EtOH: 50%/50%) containing the radiolabelled ^61^Cu-conjugated compound under study was added to 450 μL of each medium (0.9% NaCl or PBS), and the mixtures were incubated at 37 °C (T0). At different time points (T0, T0 + 1 h, T0 + 2 h, T0 + 4 h, T0 + 6 h and T0 + 12 h), aliquots were taken and measured using the HPLC methods formerly characterised (Table 4).

#### 3.4.5. Stability in Mice Serum

For stability in mice serum, 500 μL of serum was incubated with 50 μL of ^61^Cu-conjugated peptides dissolved in the final formulation, at 37 °C. At different time points (T0, T0 + 1 h, T0 + 2 h, T0 + 4 h, T0 + 6 h and T0 + 12 h), 50 μL aliquots were taken, and 150 μL of ethanol was added to precipitate the plasma proteins. The mixture was centrifuged at 3000 rpm for 10 min and the supernatant was collected and diluted in NaCl 0.9% for HPLC analysis.

## 4. Conclusions

In this study, we demonstrated that clinical amounts of ^61^Cu-based radiopharmaceuticals can be produced, under GMP, in a medical cyclotron, using liquid targets. Production yields are higher using enriched target in comparison to irradiating natural zinc. The high radionuclidic and radiochemical purity of the produced ^61^Cu-labelled radiopharmaceuticals ([^61^Cu]Cu-DOTA-NOC, [^61^Cu]Cu-DOTA-TOC and [^61^Cu]Cu-DOTA-TATE), opens the possibility for them to be used as an alternative to the current clinically used versions with gallium-68. This work serves as background for future preclinical in vitro and in vivo studies aiming at bringing copper-61 radiopharmaceuticals to the clinical setting in the near future.

## Figures and Tables

**Table 1:** Irradiation conditions applied to each target and total activity produced (GBq) at EOB.

Target	n	[HNO_3_] (M)	[Zn] (mg/mL)	I (μAh)	Irrad. Time (min)	Act. Produced (GBq)
^nat^Zn(p,α)^61^Cu	20	0.01	200	70.1 ± 0.3	180	1.84 ± 0.24
^64^Zn(p,α)^61^Cu	32	0.01	200^1^	67.4 ± 2.9	180	3.28 ± 0.41

Initial concentration before recycling. EOB: End Of Bombardment.

**Table 2:** Summary of activities and Yields (i.e., Labelling Yield and RCY) achieved in the radiopharmaceutical synthesis of [^61^Cu]Cu-DOTA-NOC, [^61^Cu]Cu-DOTA-TATE and [^61^Cu]Cu-DOTA-TOC produced from either natural or enriched zinc.

Radiopharmaceutical	Target	Process Duration (min)	Activity @EOS (GBq)	Labelling Yield (%)	RCY (%)
[^61^Cu]Cu-DOTA-NOC N = 5	Natural Zinc	32 ± 4	0.99 ± 0.16	98.48 ± 0.89	94.73 ± 3.03
[^61^Cu]Cu-DOTA-NOC N = 5	Zinc-64	38 ± 2	1.95 ± 0.21	97.72 ± 2.01	94.03 ± 1.84
[^61^Cu]Cu-DOTA-TATE N = 3	Zinc-64	37 ± 6	2.06 ± 0.08	98.61 ± 0.84	95.91 ± 1.50
[^61^Cu]Cu-DOTA-TOC N = 3	Zinc-64	38 ± 4	1.77 ± 0.12	97.87 ± 1.10	94.67 ± 1.19

RCY: radiochemical yield. EOS: End Of Synthesis.

**Table 3:** Final product specifications for [^61^Cu]Cu-DOTA-NOC, [^61^Cu]Cu-DOTA-TATE and [^61^Cu]Cu-DOTA-TOC (mean ± SD, N ≥ 3).

Production Route	^nat^Zn(p,α)^61^Cu		^64^Zn(p,α)^61^Cu	
TEST	[^61^Cu]Cu-DOTA-NOC	[^61^Cu]Cu-DOTA-NOC	[^61^Cu]Cu-DOTA-TATE	[^61^Cu]Cu-DOTA-TOC
MA (MBq/nmol)	28.93 ± 4.58	56.82 ± 6.25	52.31 ± 9.83	50.27 ± 3.40
Activity at EOS (GBq)	0.99 ± 0.16	1.95 ± 0.21	2.06 ± 0.08	1.77 ± 0.12
RCP (%)	99.48 ± 0.51	98.71 ± 0.57	99.90 ± 0.03	99.77 ± 0.16
RNP (%)	98.49 ± 0.07		99.97 ± 0.03	
Radionuclidic identity (h)	3.33 ± 0.04	3.33 ± 0.04	3.33 ± 0.04	3.33 ± 0.04
pH	3–5	3–5	3–5	3–5
Visual Inspection	Clear, Colourless	Clear, Colourless	Clear, Colourless	Clear, Colourless
Volume (mL)	5–10	5–10	5–10	5–10

MA: Molar activity. RCP: Radiochemical Purity. RNP: Radionuclidic Purity.

**Table 4:** HPLC methods for RCP determination.

	Time (min)	Mobile Phase A (Per Cent *v*/*v*)	Mobile Phase B (Per Cent *v*/*v)*
Solvents		Water/0.1% TFA	ACN/0.1% TFA
Method A	0–11	74 → 60	26 → 40
	11–12	60 → 40	40 → 60
	12–14	40	60
Method B	0–8	78	22
	8–9	78 → 40	22 → 60
	9–14	40	60

## Data Availability

Data is contained within the article and supplementary material.

## Supplementary Materials

The following supporting information can be downloaded at: https://www.mdpi.com/article/10.3390/ph15060723/s1, Table S1: Comparison of copper-61 activity produced and purified, corrected at EOB and EOP, respectively, when using non-recycled, once recycled, twice recycled, and three times recycled zinc-64 solution (mean ± SD, N = 8). Isotopic enrichment of the irradiated zinc-64 recycled solution determined by ICP-MS analysis.
