# Supplementary material for: Production of GMP-Compliant Clinical Amounts of Copper-61 Radiopharmaceuticals from Liquid Targets
Source: Pharmaceuticals (Basel). 2022 Jun 7;15(6):723. doi: 10.3390/ph15060723 (PMC9231368; doi:10.3390/ph15060723)
Supplement: Supplementary file 1 [file pharmaceuticals-15-00723-s001.zip › pharmaceuticals-1716489-supplementary.pdf]

**Table S1.** Comparison of copper-61 activity produced and purified, corrected at EOB and EOP, respectively, when using non-recycled, once recycled, twice recycled, and three times recycled zinc-64 solution (mean ± SD, N=8). Isotopic enrichment of the irradiated zinc-64 recycled solution determined by ICP-MS analysis.

| | **Activity Produced at EOB (GBq)** | **Activity Purified at EOP (GBq)** | **Zinc-64 Isotopic Enrichment (%)** |
|---|---|---|---|
| 1st Irradiation | 3.65 ± 0.18 | 2.34 ± 0.16 | 99.90 ± 0.01 |
| 2nd Irradiation | 3.43 ± 0.26 | 2.22 ± 0.19 | 99.89 ± 0.01 |
| 3rd Irradiation | 3.09 ± 0.34 | 2.09 ± 0.22 | 99.88 ± 0.01 |
| 4th Irradiation | 2.94 ± 0.41 | 2.00 ± 0.39 | 99.81 ± 0.12 |

EOB: End of Beam
EOP: End of Purification
